# Putative binding of *Piper betle* L. phytochemicals to mannitol pathway enzymes during sporulation of *Eimeria tenella*

**DOI:** 10.1016/j.vas.2026.100763

**Published:** 2026-07-10

**Authors:** Syahputra Wibowo, Penny Humaidah Hamid, Rina Ristanti, Herjuno Ari Nugroho, Maulana Malik Nashrulloh, Tri Rini Nuringtyas, Pamungkas Rizki Ferdian, R. Lia Rahadian Amalia, Tamara Munoz Caro, Nur Balqis Maulydia, Shelly Kusumarini, April Hari Wardhana, Muhammad Cahyadi, Makoto Matsubayashi

**Affiliations:** a Eijkman Research Center for Molecular Biology, National Research and Innovation Agency (BRIN), Indonesia; b Faculty of Animal Science, Universitas Sebelas Maret, Indonesia; c Tropical OneHealth and EcoHealth Institute, Indonesia; d Institute of Research and Community Service, Universitas Sebelas Maret, Indonesia; e Research Center for Applied Microbiology, National Research and Innovation Agency (BRIN), Indonesia; f Division of Biomics Research, Department of Sciences, Generasi Biologi Indonesia Foundation, Indonesia; g Research Center for Biotechnology, Graduate School, Universitas Gadjah Mada, Indonesia; h Faculty of Biology, Universitas Gadjah Mada, Indonesia; i Research Center for Applied Zoology (BRIN), Indonesia; j Escuela de Medicina Veterinaria, Facultad de Medicina Veterinaria Y Recursos Naturales, Universidad Santo Tomas, Chile; k Department of Parasitology, Faculty of Veterinary Medicine, Brawijaya University, Indonesia; l Research Center for Veterinary Science (BRIN), Indonesia; m Veterinary Medicine, Osaka Metropolitan University, Japan

**Keywords:** Coccidian, Sporogony, Metabolism, Drug-target

## Abstract

Coccidiosis, caused by *Eimeria* species, remains a major challenge to the poultry industry. The parasite transmission relies on the environmental sporogony stage during which the parasite transforms from non-infectious to infectious oocysts. This host-independent metabolism is driven by the mannitol pathway, which operates in oocysts of *Eimeria, Toxoplasma*, and *Cryptosporidium*. Our previous *in vitro* study demonstrated that *Piper betle* L. essential oil (PBEO) exhibits anticoccidial activity through oocysticidal effects and sporulation inhibition (IC₅₀ 1.31% at 72 h). Here, we performed systematic *in silico* molecular docking to evaluate ten major PBEO phytochemicals against three key *E. tenella* mannitol pathway enzymes: mannitol-1-phosphate dehydrogenase (M1PDH), mannitol-1-phosphate phosphatase (M1Pase), and hexokinase. In hexokinase enzyme, 3-allyl-6-methoxyphenyl acetate showed the strongest binding affinity (-6.2 kcal/mol), stabilized by a conventional hydrogen bond with CYS161 and a pi-sigma interaction with Leu58. For M1Pase, β-selinene and β-caryophyllene exhibited the highest affinities (-5.9 kcal/mol each) through hydrophobic alkyl networks. Next, the M1PDH receptor, β-selinene, achieved the most favourable binding (-7.4 kcal/mol), closely followed by the bicyclic sesquiterpene derivative (-7.1 kcal/mol). PBEO constituents potentially exert anti-sporulation effects by simultaneous inhibition of multiple mannitol pathway enzymes. Kyoto Encyclopedia of Genes and Genomes (KEGG) pathway mapping and analysis (map00051) confirms that these enzymes form a non-redundant metabolic unit essential for sporulation, with no bypass reactions annotated in the parasite genome. These findings support further bio-guided isolation and development of PBEO-derived compounds as anticoccidial agents targeting the environmental stage of coccidian parasites.

## Introduction

Avian coccidiosis remains one of the most economically significant diseases affecting poultry production worldwide, with global losses estimated at approximately US$13 million annually due to medication costs, mortality, reduced feed efficiency, and decreased productivity (Chapman et al., 2013; Gilbert et al., 2020; Muñoz-Gómez et al., 2025; Shirley et al., 2005). The disease is caused by apicomplexan parasites of the genus *Eimeria*, with seven species known to infect chickens (Chapman et al., 2013; López-Osorio et al., 2020). Among these, *Eimeria tenella* is recognized as the most prevalent and pathogenic species in Southeast Asia, primarily targeting the cecal epithelial cells and causing characteristic hemorrhagic lesions and bloody diarrhea (Chen et al., 2025; Hamid et al., 2018).

**Fig. 1:**
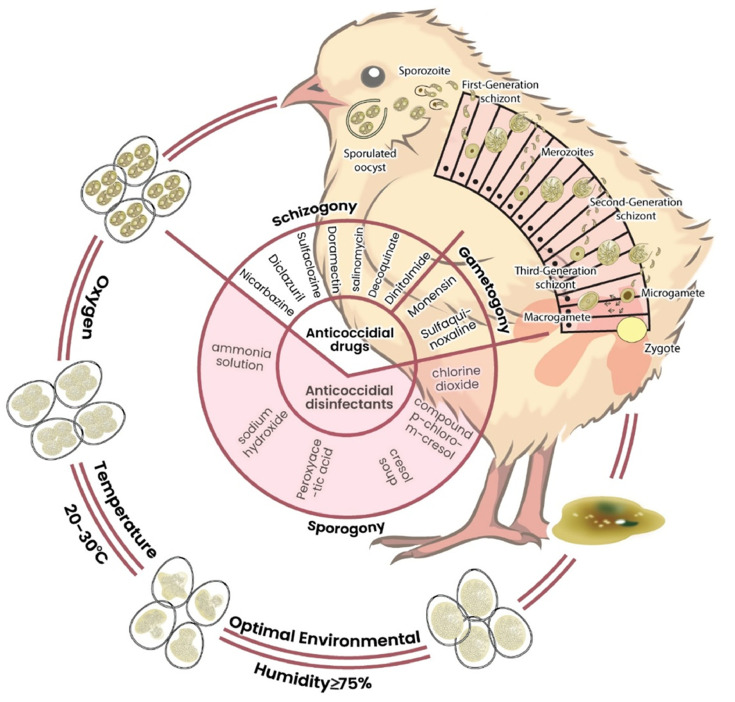
Life cycle of *Eimeria tenella* with emphasis on oocyst sporulation. Illustration of the *E. tenella* life cycle, focusing on the transformation of unsporulated oocysts excreted in feces into sporulated, infectious oocysts. The sporulation process involves morphological differentiation and metabolic activation, resulting in the formation of sporocysts containing sporozoites capable of initiating infection in the host.

The life cycle of *Eimeria* species comprises both endogenous (within the host) and exogenous (environmental) phases (Fig. 1). Following oral ingestion of sporulated oocysts, sporozoites are released and invade epithelial cells, where they undergo merogony and gametogony, producing unsporulated oocysts that are shed in feces (López-Osorio et al., 2020). These unsporulated oocysts undergo sporogony in the external environment, a critical process that transforms non-infectious oocysts into infectious sporulated oocysts containing four sporocysts, each harboring two sporozoites (Francia and Striepen, 2014; López-Osorio et al., 2020). This environmental stage is particularly vulnerable to intervention, as the parasite relies solely on its own energy reserves and is independent of the host system (Amiar et al., 2020). During sporogony, *Eimeria* parasites rely on mannitol as their primary energy source, with approximately 90% of mannitol reserves consumed within the first 15 hours of sporulation (Allocco et al., 1999). This mannitol cycle represents a distinctive metabolic pathway unique to coccidian parasites, operating exclusively during the exogenous environmental stage when oocysts develop independently of the host (Schmatz, 1989, 1997). Unsporulated *E. tenella* oocysts contain approximately 150-200 mM mannitol, constituting a substantial portion of their total carbon reserves that is rapidly depleted during critical developmental events including sporocyst formation, sporozoite differentiation, and refractile body development (Michalski et al., 1992; Schmatz, 1989, 1997). The absence of this pathway in mammalian hosts positions mannitol cycle enzymes as ideal targets for selective anticoccidial intervention with minimal host toxicity, making them attractive candidates for disrupting sporulation, breaking the transmission cycle, and preventing coccidiosis outbreaks (Allocco et al., 2001).

**Fig. 2:**
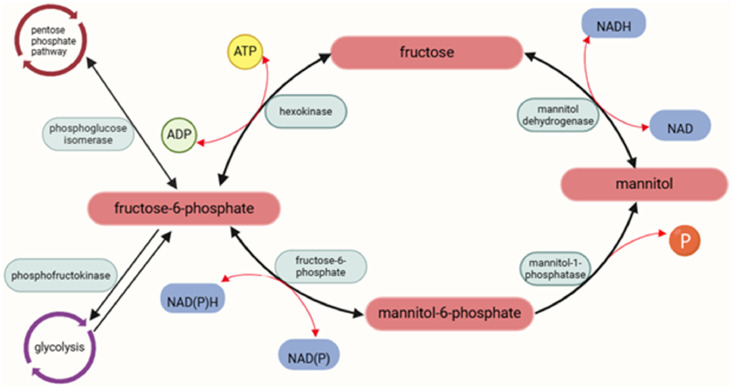
The mannitol cycle in *Eimeria tenella* (modified from Schmatz et al., 1989). This metabolic pathway operates during the sporogony process of Eimeria parasites, serving as a critical energy source when oocysts are in the external environment independent of the host. The cycle involves the conversion of fructose to mannitol and back, with key enzymes including hexokinase, phosphoglucose isomerase, and mannitol dehydrogenase. The pathway intersects with both the pentose phosphate pathway and glycolysis, utilizing ATP, NADH, and NAD(P)H as cofactors. During sporulation, approximately 90% of the mannitol reserves are consumed within the first 15 hours, highlighting the importance of this cycle for successful oocyst sporulation and subsequent transmission to susceptible hosts.

The mannitol cycle in *E. tenella* (Fig. 2) involves four key enzymes (Schmatz, 1989). Mannitol-1-phosphate dehydrogenase (M1PD) converts fructose-6-phosphate to mannitol-1-phosphate using either NADH or NADPH as a cofactor. Mannitol-1-phosphatase (M1Pase) then dephosphorylates mannitol-1-phosphate to produce free mannitol. Subsequently, mannitol dehydrogenase (MDH) oxidizes mannitol to fructose specifically using NAD⁺, and finally, hexokinase phosphorylates fructose to regenerate fructose-6-phosphate, completing the cycle. Schmatz et al. (1989) first characterized this cycle in *E. tenella* and demonstrated distinct differences from fungal mannitol cycles, particularly in coenzyme requirements: M1PD in *E. tenella* accepts both NADH and NADPH (versus a specific NADH requirement in fungi), while MDH specifically requires NAD (versus NADP in fungi) (Allocco et al., 1999; Michalski et al., 1992; Schmatz, 1989).

The urgent need for coccidiosis interventions is underscored by the widespread emergence of coccidiostat resistance. For decades, the poultry industry has relied heavily on a limited arsenal of synthetic coccidiostats (e.g., ionophores, chemical derivatives) to control *Eimeria* infections. However, prolonged and intensive use has led to the selection of resistant parasite strains across multiple species, severely compromising the efficacy of many conventional drugs (Martins et al., 2022). Recent field surveillance has confirmed the continued decline in susceptibility to standard anticoccidials, necessitating higher doses or rotational programs that are often unsustainable and may increase production costs (Estensmo et al., 2026). Our recent investigations have demonstrated that *Piper betle* L. essential oil (PBEO) exhibits significant anticoccidial effects against *E. tenella* oocysts through two primary mechanisms: oocysticidal activity by disintegrating oocyst walls and inhibition of the sporulation process (Ristanti et al., 2024). At concentrations as low as 0.04%, PBEO significantly reduced sporulated oocysts to 71.67%, with an IC_50_ of 1.31% at 72 h post-incubation. Gas chromatography-mass spectrometry (GC-MS) analysis identified the primary constituents of PBEO, including eugenol, and other key compounds, collectively constituting 96% of the oil (Ristanti et al., 2024). However, the molecular mechanisms underlying PBEO's inhibition of sporulation remain poorly understood.

Despite the established importance of this pathway and our recent in vitro evidence that PBEO inhibits sporulation (Ristanti et al., 2024), the specific molecular interactions between PBEO phytochemicals and mannitol cycle enzymes remain completely unexplored. Therefore, in this study, we performed systematic *in silico* molecular docking to evaluate ten major PBEO constituents against three key *E. tenella* mannitol pathway enzymes: mannitol-1-phosphate dehydrogenase (M1PDH), mannitol-1-phosphate phosphatase (M1Pase), and hexokinase. To establish the metabolic context of these targets, we mapped the mannitol cycle enzymes to the Kyoto Encyclopedia of Genes and Genomes (KEGG) pathway, confirming their integration within the broader carbohydrate metabolism network and their essential role during parasite sporulation. By targeting enzymes critical for energy metabolism during the environmental stage of the parasite, we aim to elucidate the possible mechanisms through which PBEO constituents may offer mechanistic insights into the molecular interactions underlying PBEO's anticoccidial activity and support further bioactivity-guided fractionation, and isolation of compounds targeting specific enzymes involved in the sporogony process of coccidian parasites.

## Molecular docking modelling

### Data mining

The sequences of the first committed enzymes of the mannitol pathway were retrieved from public databases: mannitol‑1‑phosphate dehydrogenase (GenBank accession AAD02688.1) from the NIH database, while mannitol‑1‑phosphate phosphatase (UniProt O43980) and hexokinase (UniProt U6KUE1) were obtained from the UniProt database (https://www.uniprot.org). Three‑dimensional models were generated using AlphaFold predictions and subsequently refined with GalaxyRefine (https://galaxy.seoklab.org/). Model quality was assessed with MolProbity (https://molprobity.biochem.duke.edu/); all final models exhibited >95% of residues in allowed regions of the Ramachandran plot. All ligands were sourced from the PubChem database (https://pubchem.ncbi.nlm.nih.gov/). The ten phytochemicals subjected to docking were selected on the basis of our previous gas chromatography-mass spectrometry (GC-MS) profile of *Piper betle* L. essential oil reported in Ristanti et al. (2024), in which these compounds collectively accounted for ∼96% of the total oil composition and represented the most abundant constituents detected. This selection therefore reflects the dominant chemical components of PBEO most likely to drive its observed anticoccidial activity.

### Reference inhibitor docking for comparative benchmarking

Nitrophenide, also known as Megasul™ (PubChem CID: 10842), was selected as a common reference compound because it has previously been reported to block *E. tenella* development through inhibition of the mannitol-cycle enzyme mannitol-1-phosphate dehydrogenase (M1PDH) (Allocco et al., 2001). The nitrophenide structure was retrieved from PubChem and docked against M1PDH, hexokinase, and M1Pase using the same parameters applied to PBEO-derived compounds, thereby providing a comparative benchmark for interpreting the predicted binding affinities of the PBEO phytochemicals.

### Protein analysis

Prior to molecular docking, the three-dimensional structures of M1Pase, M1PDH, and hexokinase were subjected to energy minimization and refinement using the GalaxyRefine web server (https://galaxy.seoklab.org/) to optimize side-chain conformations and overall stereochemistry. The quality of the refined models was evaluated with MolProbity (https://molprobity.biochem.duke.edu/) through Ramachandran plot analysis.

### Molecular docking analysis

Molecular docking was performed using a blind docking method that incorporated cavity detection guidance (https://cadd.labshare.cn/cb-dock2/index.php) (Liu et al., 2022; Maulydia et al., 2026). The outcomes were visualized utilizing Discovery Studio 2021 Client 21.1 (Dassault Systemes, France) (Manurung et al., 2026).

### KEGG pathway analysis

The mannitol metabolic pathway was mapped using KEGG database (https://www.genome.jp/kegg/). To confirm the metabolic integration of the three target enzymes, we mapped their Enzyme Commission (EC) numbers onto the KEGG Fructose and mannose metabolism pathway (map00051). The following EC numbers were used: mannitol-1-phosphate dehydrogenase (M1PDH; EC 1.1.1.17), mannitol-1-phosphate phosphatase (M1Pase; EC 3.1.3.22), and hexokinase (EC 2.7.1.1). Pathway reconstruction was performed using the KEGG Mapper tool (https://www.genome.jp/kegg/mapper/). The resulting map was visually inspected to identify the connectivity between the three enzymes and to detect potential alternative or bypass reactions.

## Results

### Protein analysis

**Fig. 3:**
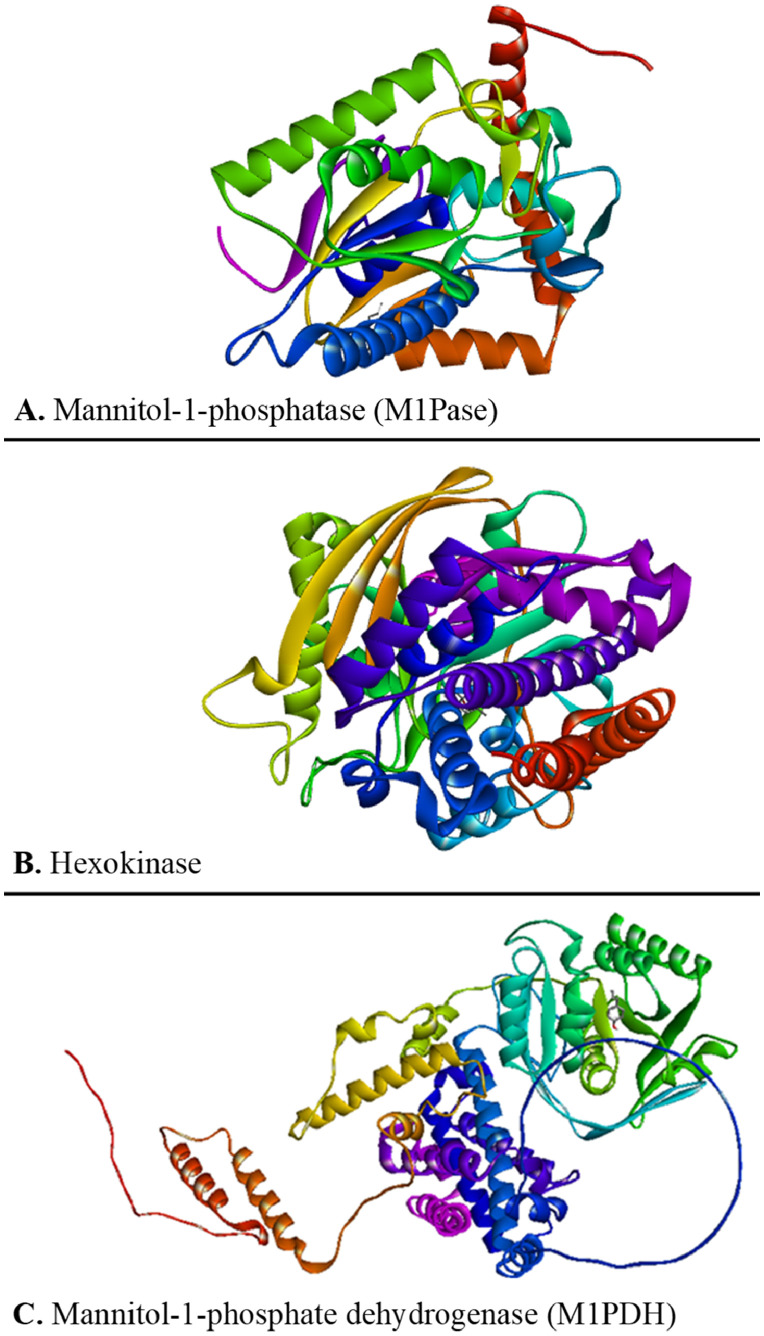
The mannitol cycle comprises three enzymes with distinct catalytic functions: **A.** mannitol-1-phosphatase (M1Pase), a highly specific phosphatase that generates free mannitol; **B.** hexokinase, which phosphorylates fructose to regenerate fructose-6-phosphate, completing the cycle; and **C.** mannitol-1-phosphate dehydrogenase (M1PDH), which initiates the cycle by reducing fructose-6-phosphate to mannitol-1-phosphate using NADH or NADPH.

All three refined models exhibited >95% of residues in allowed regions, with the majority occupying the most favourable core regions and no residues in disallowed conformations. These values indicate that the refined structures possess excellent stereochemical quality, providing a reliable basis for subsequent docking simulations and interaction analyses. The 3-dimentional structure of these receptors in this study can be seen in Fig. 3.

### Molecular docking simulation

#### Mannitol-1-phosphate (M1Pase)

**Fig. 4:**
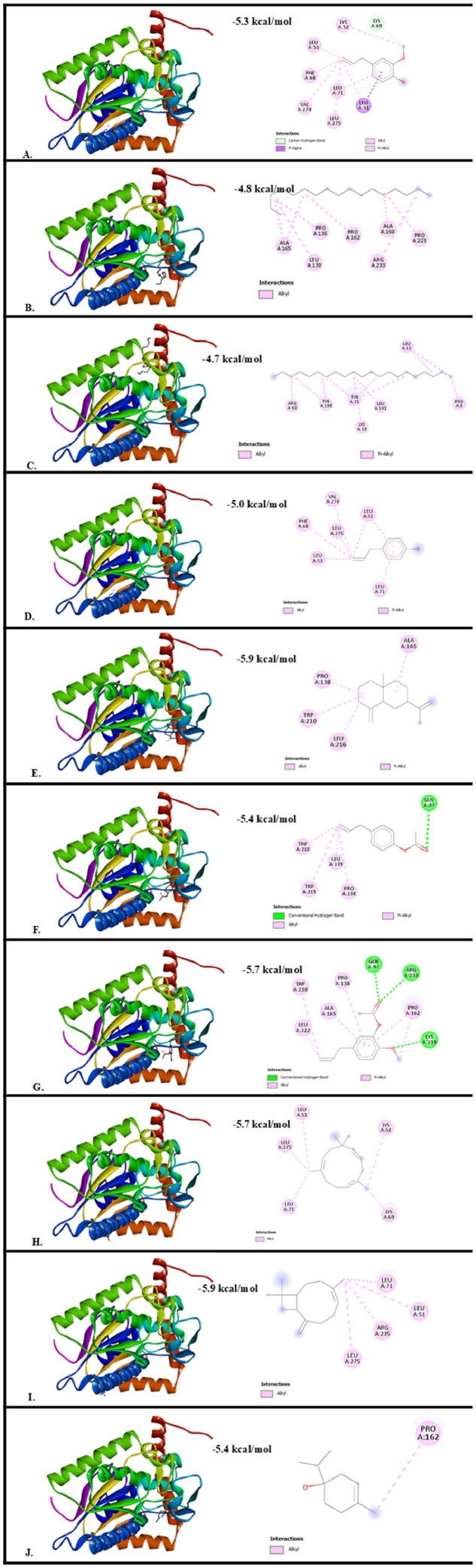
The predicted binding modes from Mannitol-1-phosphate (M1Pase) with **A**. Eugenol (-5.3 kcal/mol), **B.***n*-Heptadecane (-4.8 kcal/mol), **C.***n*-Heneicosane (-4.7 kcal/mol), **D.** Chavicol (-5.0 kcal/mol), E. β-Selinene (-5.9 kcal/mol), **F.** 4-Allylphenyl acetate (-5.4 kcal/mol), **G.** 3-Allyl-6-methoxyphenyl acetate (-5.7 kcal/mol), **H.** Humulene (-5.7 kcal/mol), **I.** β-Caryophyllene (-5.9 kcal/mol), **J.** (-)-Terpimen-4-ol (-5.4 kcal/mol).

Based on the interaction analyses and binding affinity values, β-selinene and β-caryophyllene emerged as the most effective M1Pase ligands, each with a binding affinity of -5.9 kcal/mol, reflecting stable and favourable interactions with the protein (Table 1). The former secures its position through a synergistic combination of alkyl and pi-alkyl interactions, engaging alanine (Ala A:165), proline (Pro A:138), leucine (Leu A:216), and tryptophan (Trp A:210), allowing its hydrophobic regions to align closely with the nonpolar side chains of these residues for a snug, energetically favourable fit. The latter relies on multiple hydrophobic alkyl contacts with leucine (Leu51, Leu71, and Leu275) and arginine (Arg235), creating a similarly robust anchoring network that underscores the importance of hydrophobic packing in this binding pocket. Across the entire ligand set, hydrophobic interactions, predominantly alkyl and pi-alkyl contacts, serve as the dominant stabilizing force with residues such as Leu51, Leu71, Leu275, Ala165, Pro138, and Trp210 recurring frequently, strongly suggesting the presence of a conserved nonpolar cavity within M1Pase that accommodates a variety of terpene and phenylpropanoid scaffolds (Fig. 4).

**Table 1:** Molecular docking analysis of 10 dominant substances of *P. betle* essential oil (PBEO), including binding affinities and predicted interaction residues. Binding energies (kcal/mol) indicate the strength of ligand-receptor interactions, while interacting amino acid residues are categorized based on hydrophobic interactions (HI), and polar hydrogen interactions (PHI).

Receptor	Ligand	Binding free-energy value (kcal/mol)	Type of interaction	Amino acids involved
Mannitol-1-phosphate dehydrogenase (M1PDH)	Nitrophenide	-6.9	HI	Gln A:206 Arg A:207
			PHI	Leu A:205 Ala A:279 Ala A:300 Leu A:304
	Eugenol	–5.6	HI	Thr A:273 Asp A:275
			PHI	Ala A:279 Asp A:275 Ala A:270 Leu A:205 Leu A:293
	(–)-Terpinen-4-ol	–5.8	HI	Ala A:279 Leu A:205 Phe A:180 Ala A:300 Leu A:304 Ala A:290
			PHI	–
	Chavicol	–5.6	HI	Leu A:304 Ala A:279 Ala A:300
			PHI	Ala A:279 Ala A:300
	4-Allylphenyl acetate	–5.9	HI	Leu A:304 Leu A:205 Ala A:279
			PHI	Gly181
	β-Caryophyllene	–7.1	HI	Leu A:304 Ala A:300 Leu A:293 Leu A:205 Ala A:301 Phe A:180 Ala A:279 Ala A:270
			PHI	–
	Humulene	–6.6	HI	Phe A:180 Leu A:293 Ala A:279 Leu A:205 Arg A:207 Ala A:300
			PHI	–
	*n*-Heptadecane	–5.0	HI	Leu A:293 Ala A:300 Arg A:207 Leu A:205 Ala A:279 Leu A:304
			PHI	–
	*n*-Heneicosane	–4.9	HI	Trp A:511 Pro A:152 Leu A:151 Phe A:95 Ala A:115 Leu A:111 Ile A:114 Leu A:143 His A:401
			PHI	–
	β -selinene	–7.4	HI	Leu A:205 Arg A:207 Leu A:293 Ala A:279 Ala A:300 Leu A:304
			PHI	–
	3-Allyl-6-methoxyphenyl acetate	–6.2	HI	Arg A:207 Leu A:293 Leu A:205 Ala A:279 Leu A:304 Ala A:300
			PHI	–
M1Pase	Nitrophenide	-6.3	HI	Asn A:88 Arg A:92 Ser A:266
			PHI	–
	Eugenol	-5.3	HI	Leu A:51 Lys A:52 Leu A:53 Phe A:68 Leu A:71 Val A:274 Leu A:275
			PHI	Lys A:69
	*n*-Heptadecane	-4.8	HI	Pro A:138 Leu A:139 Pro A:162 Ala A:165 Ala A:168 Pro A:223 Arg A:233
			PHI	-
	*n*-Heneicosane	-4.7	HI	Pro A:8 Leu A:11 Tyr A:15 Lys A:19 Arg A:92 Tyr A:190 Leu A:191
			PHI	-
	Chavicol	-5.0	HI	Leu A:51 Leu A:53 Phe A:68 Leu A:71 Val A:274 Leu A:275
			PHI	-
	β-selinene	-5.9	HI	Pro A:138 Ala A:165 Trp A:210 Leu A: 216
			PHI	-
	4-Allylphenyl acetate	-5.4	HI	Pro A:138 Leu A:239 Trp A:210 Trp A:215
			PHI	Gln A:97
	3-Allyl-6-methoxyphenyl acetate	-5.7	HI	Pro A:138 Pro A:162 Ala A:165 Trp A:210 Leu A:222
			PHI	Gln A:97 Lys A:219 Arg A:233
	Humulene	-5.7	HI	Leu A:51 Lys A:52 Lys A:69 Leu A:71 Leu A: 275
			PHI	-
	β-Caryophyllene	-5.9	HI	Leu A:51 Leu A:71 Arg A:235 Leu A:275
			PHI	-
	(-)-Terpinen-4-ol	-5.4	HI	Pro A:162
			PHI	-
Hexokinase	Nitrophenide	-6.8	HI	Asn A:479 Asn A:510 Gln A:558
			PHI	Pro A:424 Glu A:531
	Eugenol	-5.3	HI	Leu A:275 Asp A:285 Leu A:295 Lys A:298 Met A:299
			PHI	Thr A:277
	*n*-Heptadecane	-4.7	HI	Leu A:46 Leu A:58 Pro A:160 Val A:162
			PHI	-
	*n*-Heneicosane	-4.9	HI	Leu A:46 Trp A:48 Leu A:58 Cys A:161 Val A:162
			PHI	-
	Chavicol	-5.5	HI	Asp A: 285 Leu A:295 Lys A:298 Met A:199
			PHI	Thr A:277 Lys A:292
	β-selinene	-6.1	HI	Cys A:161
			PHI	-
	4-Allylphenyl acetate	-6.1	HI	Leu A:46 Leu A:58
			PHI	Asn A:215
	3-Allyl-6-methoxyphenyl acetate	-6.2	HI	Leu A:46 Leu A:58
			PHI	Cys A:161
	Humulene	-5.7	HI	Leu A:58 Cys A:161
			PHI	
	β-Caryophyllene	-6.1	HI	Ile A:65 Pro A:234 Trp A:250
			PHI	-
	(-)-Terpinen-4-ol	-5.7	HI	Leu A:58
			PHI	-

In contrast, polar hydrogen bonds play a secondary, context-dependent role, appearing only in a subset of compounds: eugenol via carbon-hydrogen bonds with Lys A:69 and Leu A:205; 4-allylphenyl acetate through a conventional hydrogen bond with Gln A:97; and 3-allyl-6-methoxyphenyl acetate engaging Gln97, Arg233, and Lys219. Even among the aliphatic hydrocarbons, *n*-heptadecane and *n*-heneicosane, which rely exclusively on alkyl interactions with residues including Ala165, Ala168, Leu139, and Pro138, exhibited the weakest binding affinities (-5.0 and -4.9 kcal/mol, respectively). Their linear, flexible chains may be less effectively accommodated within the confined hydrophobic pocket compared to the more rigid, compact frameworks of the sesquiterpene derivatives, potentially incurring an entropic penalty that offsets favourable van der Waals contacts. These findings not only identify the two sesquiterpene derivatives as promising lead scaffolds but also delineate a conserved hydrophobic hotspot, centred around Leu51, Leu71, Leu275, Ala165, and Pro138, that serves as an anchor for high-affinity binding. Future optimization efforts should aim to preserve these core hydrophobic contacts while strategically introducing polar functionalities that engage nearby residues such as Gln97, Arg233, or Lys219, thereby harnessing both hydrophobic and polar contributions to achieve enhanced selectivity and inhibitory potency against M1Pase.

#### Hexokinase

**Fig. 5:**
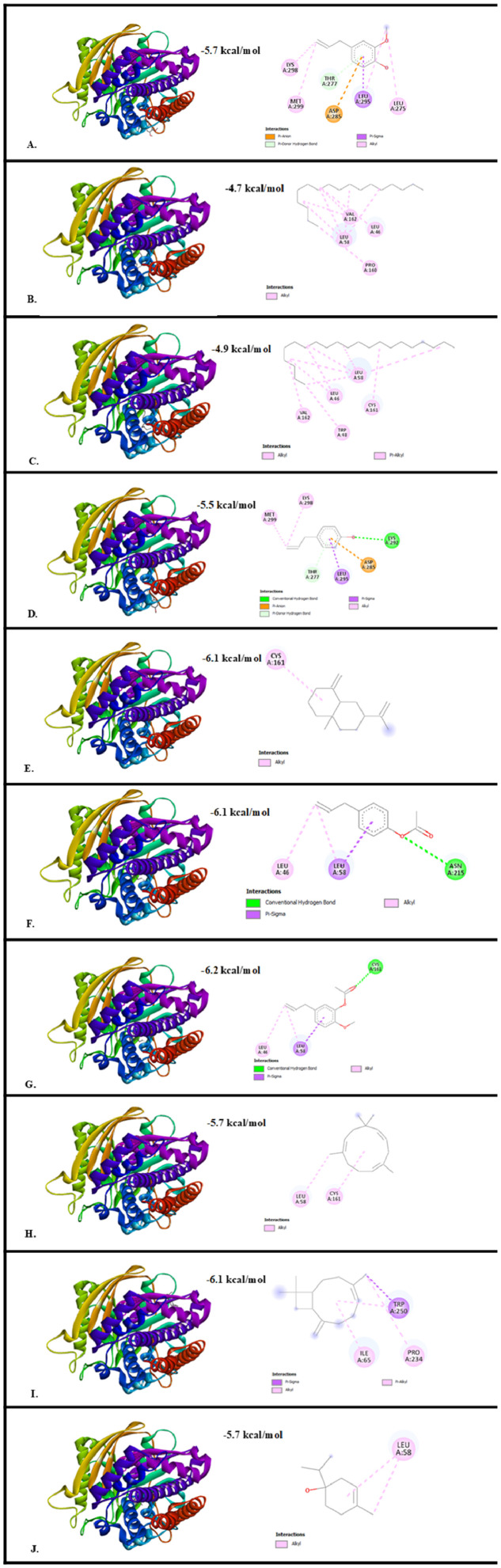
The predicted binding modes of Hexokinase receptors with **A.** Eugenol (-5.7 kcal/mol), **B.***n*-Heptadecane (-4.7 kcal/mol), **C.***n*-Heneicosane (-4.9 kcal/mol), **D.** Chavicol (-5.5 kcal/mol), **E.** β-Selinene (-6.1 kcal/mol), **F.** 4-Allylphenyl acetate (-6.1 kcal/mol), **G.** 3-Allyl-6-methoxyphenyl acetate (-6.2 kcal/mol), **H.** Humulene (-5.7 kcal/mol), **I.** β-Caryophyllene (-6.1 kcal/mol), **J.** (-)-Terpimen-4-ol (-5.7 kcal/mol).

**Fig. 6:**
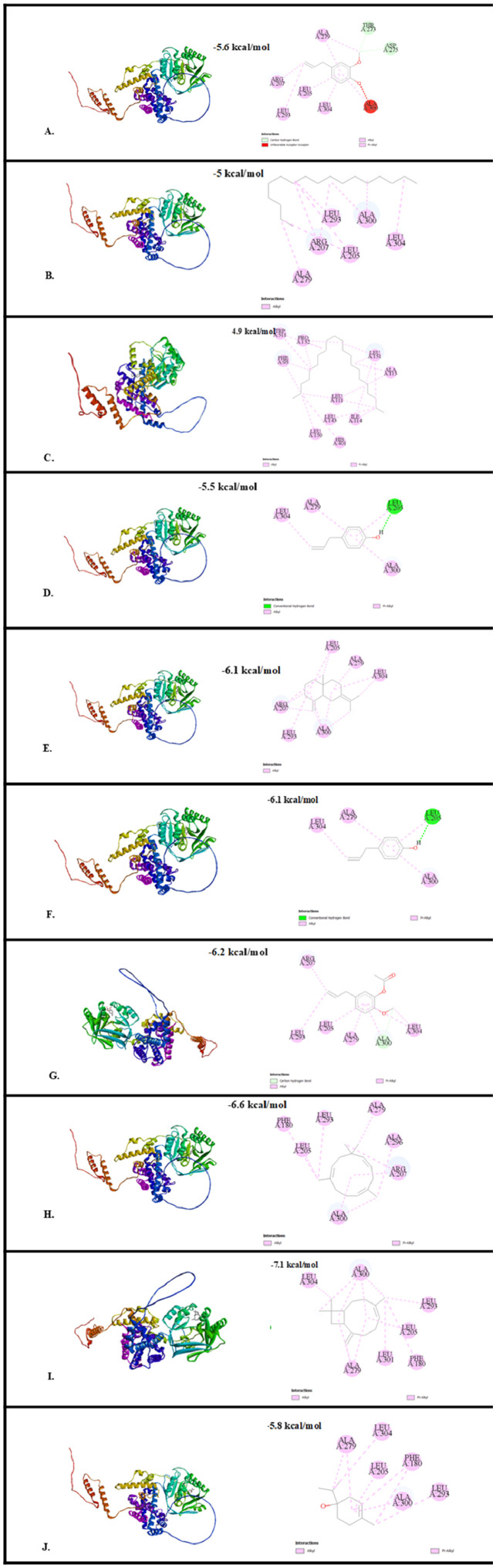
The predicted binding modes of Mannitol-1-phosphate dehydrogenase (M1PDH) with **A.** Eugenol (-5.6 kcal/mol), **B.***n*-Heptadecane (-5 kcal/mol), **C.***n*-Heneicosane (-4.9 kcal/mol), **D.** Chavicol (-5.6 kcal/mol), **E.** β-Selinene (-7.4 kcal/mol), **F.** 4-Allylphenyl acetate (-5.9 kcal/mol), **G.** 3-Allyl-6-methoxyphenyl acetate (-6.2 kcal/mol), **H.** Humulene (-6.6 kcal/mol), **I.** β-Caryophyllene (-7.1 kcal/mol), **J.** (-)-Terpinen-4-ol (-5.8 kcal/mol).

Based on the interaction analyses and the reported binding affinity, 3-Allyl-6-methoxyphenyl acetate emerged as the most effective hexokinase ligand, with a binding energy of -6.2 kcal/mol, reflecting the strongest and most stable interaction among the tested compounds. Its binding is underpinned by a complementary network of polar and hydrophobic contacts: a conventional hydrogen bond with cysteine (Cys161) provides directional stabilization, while a pi‑sigma interaction with leucine (Leu58) aligns the ligand’s aromatic ring with the protein’s hydrophobic environment, further reinforced by alkyl interactions from leucine residues (Leu46 and Leu58). This balanced combination of polar and nonpolar contacts distinguishes it from other ligands that rely more heavily on a single interaction type. Across the results, residues such as Leu58, Cys161, and Leu46 recur frequently, appearing in complexes with n‑heptadecane, humulene, and (–)‑terpinen‑4‑ol, suggesting they form a conserved hydrophobic subpocket that accommodates a range of structures. Notably, eugenol and 4‑(2‑propenyl)‑phenol engage additional polar motifs, including pi‑anion interactions with aspartic acid (Asp285) and pi‑donor hydrogen bonds with threonine (Thr277), yet neither achieves the same overall affinity as 3‑allyl‑6‑methoxyphenyl acetate (Fig. 5). Even the weaker binders, such as *n*‑heptadecane and *n*‑heneicosane, rely exclusively on alkyl contacts, underscoring that while hydrophobic packing is critical, the inclusion of strategically placed polar groups can significantly enhance binding. Taken together, these findings identify 3‑allyl‑6‑methoxyphenyl acetate as a promising lead for hexokinase inhibition and highlight the recurring involvement of Leu58, Cys161, and Leu46 as key anchor points. Future optimization may focus on preserving these core hydrophobic interactions while fine‑tuning the polar functionalities to further improve affinity and selectivity. The corresponding 2D interaction diagram for this top-ranked pair is shown in Fig. 5G, which illustrates the conventional hydrogen bond with Cys161 (green dashed line), the pi-sigma interaction with Leu58 (purple dashed line) and the surrounding alkyl and pi-alkyl contacts that together stabilize the ligand within the hexokinase pocket. Equivalent diagrams for the top-ranked compounds binding M1PDH (β-selinene; Fig. 6E) and M1Pase (β-selinene and β-caryophyllene; Fig. 4E and I) are also presented to allow the reader to visualize the key residue-level interactions for the most promising compound-enzyme pairs without needing to cross-reference all panels.

#### Mannitol-1-phosphate dehydrogenase (M1PDH)

Molecular docking of ten ligands against M1PDH revealed a range of binding free energies from -4.9 to -7.4 kcal/mol, with the most favourable binding observed for β-selinene (-7.4 kcal/mol) and β-caryophyllene (-7.1 kcal/mol). These values suggest moderate to strong affinity, comparable to typical small‑molecule inhibitors of related dehydrogenases. Analysis of the interaction patterns (Table 1) shows that the majority of ligands engage M1PDH through hydrophobic contacts (alkyl and pi‑alkyl interactions) with residues lining the putative binding pocket. Notably, residues Ala279, Leu205, Leu293, Ala300 and Leu304 appear repeatedly across different ligands, indicating a conserved hydrophobic sub pocket that may be critical for ligand recognition. The strong binding of β-selinene and the bicyclic sesquiterpene derivative can be attributed to their extended hydrophobic surfaces, which allow extensive van der Waals contacts with this cluster of aliphatic and aromatic side chains. Only three compounds, namely eugenol, 4‑(2‑propenyl)‑phenol, and 4‑allylphenyl acetate, formed polar hydrogen interactions such as conventional or carbon‑hydrogen bonds with the protein. Interestingly, *n*‑heptadecane and *n*‑heneicosane, both straight‑chain alkanes, relied exclusively on alkyl interactions and showed the weakest binding among the tested set (-5.0 and -4.9 kcal/mol, respectively) (Fig. 6). Their linear, flexible structure may prevent optimal accommodation within the relatively constrained hydrophobic pocket, leading to entropic penalties and reduced affinity. These results indicate that M1PDH possesses a hydrophobic binding region that can accommodate a variety of terpenes and phenylpropanoids. The recurring involvement of Ala279, Leu205, Leu293, Ala300 and Leu304 highlights these residues as potential hot spots for inhibitor design. Future structure‑activity relationship studies could focus on introducing hydrogen‑bond donors or acceptors that engage the polar residues identified here (e.g., ASP275, GLY181) while maintaining the hydrophobic core interactions, potentially yielding more potent and selective M1PDH inhibitors.

#### PBEO phytochemical binding affinities against nitrophenide

**Fig. 7:**
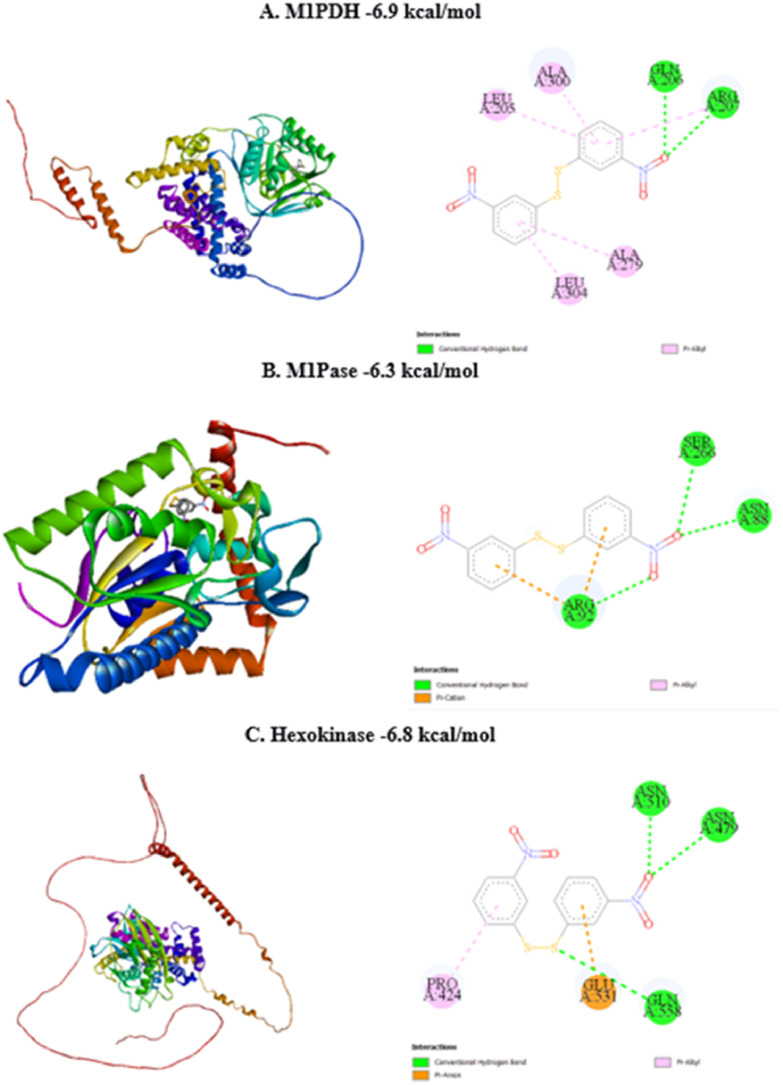
Reference docking of nitrophenide against selected *E. tenella* metabolic enzymes. **A**. Predicted binding interaction of nitrophenide with mannitol-1-phosphate dehydrogenase (M1PDH), showing a reference binding affinity of -6.9 kcal/mol. **B**. Predicted binding interaction of nitrophenide with mannitol-1-phosphatase (M1Pase), showing a reference binding affinity of -6.3 kcal/mol. **C**. Predicted binding interaction of nitrophenide with hexokinase, showing a reference binding affinity of -6.8 kcal/mol. These docking results were used as comparative benchmarks for interpreting the predicted binding affinities of PBEO phytochemicals against the corresponding metabolic targets.

The docking of nitrophenide against the three selected *E. tenella* metabolic enzymes yielded reference binding affinities of -6.9 kcal/mol for M1PDH, -6.8 kcal/mol for hexokinase, and -6.3 kcal/mol for M1Pase (Fig. 7.**A, B, C**). These energetic rankings can be rationalised by the distinct non-covalent interaction patterns observed in each enzyme-ligand complex. For M1PDH, the strongest affinity is achieved through a synergistic combination of conventional hydrogen bonds involving Gln A:206 and Arg A:207, together with a pi‑alkyl interaction with LEU A:304. The dual polar anchor provided by glutamine and arginine, complemented by hydrophobic packing from leucine, creates a particularly favourable binding environment. Hexokinase shows a nearly equivalent affinity (-6.8 kcal/mol) due to a different but equally effective interaction ensemble: a conventional hydrogen bond with Asn A:510, a pi‑anion interaction with Glu A:531, and a pi‑alkyl contact with Pro A:424. The electrostatic/quadrupole stabilisation of the Pi‑anion term compensates for the absence of a second hydrogen bond, keeping the free energy close to that of M1PDH. In contrast, M1Pase exhibits the weakest binding (-6.3 kcal/mol), which can be traced to its interaction pattern: conventional hydrogen bonds with Ser A:266 and Asn A:88, plus a pi‑cation interaction with Arg A:92. Although the Pi‑Cation contact contributes some stabilisation, the lack of extended hydrophobic (Pi‑Alkyl) interactions and the reliance on smaller, less polarisable hydrogen‑bond donors (serine and asparagine) reduce the overall binding enthalpy.

### KEGG pathway analysis of mannitol metabolism

**Fig. 8:**
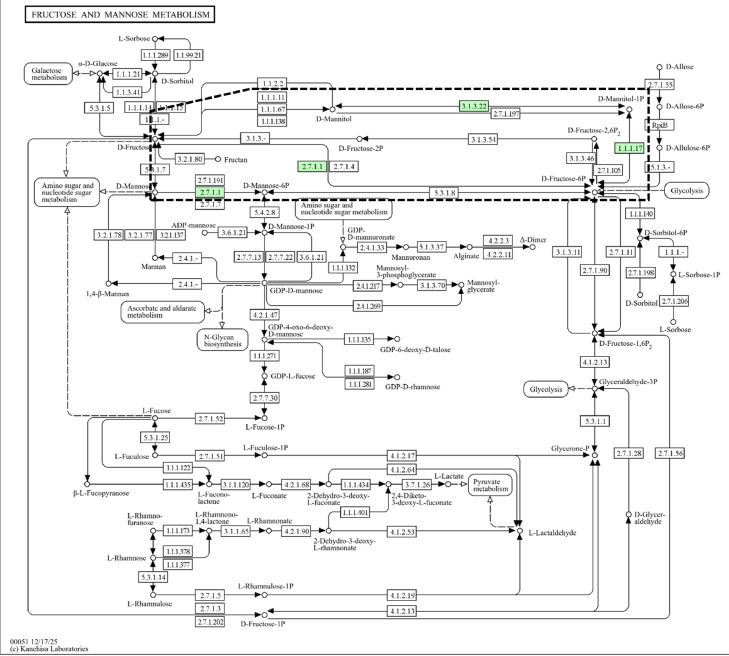
KEGG pathway map of fructose and umannose metabolism (map00051) showing the mannitol cycle enzymes. The three target enzymes are highlighted in green: mannitol-1-phosphate dehydrogenase (M1PDH, EC 1.1.1.17), mannitol-1-phosphate phosphatase (M1Pase, EC 3.1.3.22), and hexokinase (EC 2.7.1.1). The dashed box delineates the mannitol cycle, which converts fructose-6-phosphate → mannitol-1-phosphate → mannitol → fructose → fructose-6-phosphate. The pathway intersects with glycolysis (upper right) and the pentose phosphate pathway (upper left).

To verify that the three target enzymes form an integrated metabolic unit, we mapped their EC numbers onto the KEGG Fructose and mannose metabolism pathway (map00051) (Kanehisa and Goto, 2000). As shown in Fig. 8, all three enzymes were successfully located within the pathway. M1PDH (EC 1.1.1.17) catalyzes the conversion of fructose-6-phosphate to mannitol-1-phosphate. M1Pase (EC 3.1.3.22) subsequently dephosphorylates mannitol-1-phosphate to free mannitol. Hexokinase (EC 2.7.1.1) completes the cycle by phosphorylating fructose to regenerate fructose-6-phosphate.

Importantly, the KEGG map revealed that no alternative enzymes capable of bypassing these three steps are annotated in the *Eimeria tenella* metabolic repertoire within the database. The mannitol cycle was found to intersect directly with upper glycolysis (at fructose-6-phosphate) and the pentose phosphate pathway (via fructose), indicating that disruption of any of these three enzymes would likely affect broader carbon metabolism. The complete pathway visualization, with the mannitol cycle highlighted, is presented in Fig. 8.

## Discussion

The mannitol cycle is not unique to *Eimeria*. It also operates in oocysts of other apicomplexans such as *Toxoplasma gondii* and *Cryptosporidium parvum*. Mannitol serves as the main energy reserve for sporulation, comprising approximately 25% of the dry weight of unsporulated oocysts and dropping to 3% after sporulation (Allocco et al., 1999; Michalski et al., 1992; Schmatz, 1989) . Among the ten possible hexitols, only D-mannitol, D-sorbitol (D-glucitol), and galactitol occur naturally. In brown algae (Phaeophyceae), D-mannitol is a major carbon storage compound (20-30% dry weight) and also functions as an osmoprotectant and antioxidant; recent biochemical studies have further characterized the mannitol-1-phosphatase enzymes responsible for its biosynthesis in these organisms (Le Strat et al., 2023). Notably, even recent studies on mannitol utilization in engineered yeast strains have highlighted that genes involved in mannitol uptake and metabolism remain incompletely characterized, underscoring the ongoing gaps in our understanding of fungal mannitol pathways (Szczepańczyk et al., 2025). Importantly, mammalian hosts lack the ability to synthesize or degrade D-mannitol, making the mannitol cycle enzymes highly attractive targets for selective anticoccidial drug development.

The three target enzymes (M1PDH, M1Pase, and hexokinase) form an integrated and non‑redundant metabolic unit, in the KEGG Fructose and mannose metabolism pathway (map00051). This analysis revealed that the three enzymes catalyze sequential reactions that form a closed metabolic loop: fructose‑6‑phosphate→ mannitol‑1‑phosphate→ mannitol→ fructose→ fructose‑6‑phosphate (Fig. 8). Critically, the KEGG map showed that no alternative enzymes capable of bypassing these steps are annotated in the *E. tenella* genome, supporting the essential, non‑redundant role of M1PDH, M1Pase, and hexokinase during sporulation. Furthermore, the pathway intersects directly with upper glycolysis (at fructose‑6‑phosphate) and the pentose phosphate pathway (via fructose), indicating that disruption of any of these three enzymes would likely impair not only mannitol mobilization but also broader carbon metabolism.

Our findings can be contextualized against previously characterized inhibitors of the *Eimeria* mannitol cycle. Allocco et al. (2001) demonstrated that nitrophenide (Megasul™) inhibits *E. tenella* development by blocking M1PDH, with an IC₅₀ in the low micromolar range. The binding affinity of β-selinene (-7.4 kcal/mol) compares favorably with computational predictions for nitrophenide, although direct experimental comparison is needed. Unlike nitrophenide, which is a synthetic small molecule, β-selinene is a natural product with potential advantages in biodegradability and lower environmental persistence. Additionally, the multi-target activity of PBEO constituents, simultaneously affecting M1PDH, M1Pase, and hexokinase, contrasts with the single-target mechanism of most synthetic coccidiostats. This polypharmacology may confer a higher barrier to resistance development, as parasites would require concurrent mutations in multiple genes to overcome inhibition.

The mannitol cycle is active only during sporogony, the environmental stage when oocysts develop outside the host. During merogony (asexual replication within host epithelial cells), *Eimeria* relies on distinct metabolic pathways. The parasite scavenges glucose, amino acids, lipids, and nucleotides from the host cytoplasm, with glycolysis serving as the predominant energy-generating pathway (Amiar et al., 2020; Hamid et al., 2015; Piro et al., 2021). For example, *Eimeria* lacks a functional tricarboxylic acid (TCA) cycle and oxidative phosphorylation, instead converting pyruvate to lactate via lactate dehydrogenase (Denton, 1996). This reliance on glycolysis during intracellular stages has been further corroborated by recent transcriptomic and metabolomic analyses, which identified significant differential expression of key glycolytic enzymes between drug-sensitive and drug-resistant strains of *E. tenella* (Yu et al., 2025; Zhao et al., 2024). Additionally, the parasite possesses a type II fatty acid synthesis (FAS II) pathway localized within its apicoplast, which produces fatty acids for membrane biosynthesis during intracellular replication (Piro et al., 2021; Zhu, 2004). However, recent studies on related apicomplexans suggest that the functional importance of the apicoplast FAS II pathway may be context-dependent; in *Plasmodium falciparum*, for instance, FASII has been shown to be non-functional under certain conditions, highlighting the need for direct validation in *Eimeria* (Chen et al., 2024; Ouji et al., 2024).

Importantly, the mannitol cycle is not active during merogony; mannitol is synthesized only during late gametogony and stored within developing oocysts as an energy reserve for sporulation (Schmatz, 1997). This developmental stage-specific metabolic switching has been further illuminated by recent comparative proteomic analyses across *E. tenella* developmental stages, which revealed distinct protein expression profiles for unsporulated oocysts, sporulated oocysts, sporozoites, and second-generation merozoites (Ma et al., 2024). Notably, proteins involved in the electron transport chain and oxidative phosphorylation were significantly enriched in sporozoites, while the mannitol cycle enzymes showed stage-specific expression patterns consistent with their role in sporogony (Ma et al., 2024).

This developmental stage-specific metabolic switching has profound implications for drug targeting. Compounds that inhibit the mannitol cycle would selectively disrupt sporogony without affecting the merogony stage within the host. Such "transmission-blocking" anticoccidials would prevent environmental contamination with infectious oocysts while allowing the host immune system to clear existing infections. This strategy differs fundamentally from conventional coccidiostats, which target intracellular stages and exert direct selective pressure on replicating parasites, a key driver of resistance emergence. The resistance crisis has intensified in recent years, with widespread reports of reduced efficacy of both ionophores (e.g., maduramicin and salinomycin) and synthetic compounds (e.g., diclazuril) across multiple *Eimeria* species (Yu et al., 2025). Recent field surveillance from the Kashmir region of India documented partial resistance to amprolium and sulphaquinoxaline, with four *Eimeria* species persisting post-treatment, underscoring the urgent need for novel control strategies (Iqbal et al., 2025). Similarly, genomic and transcriptomic analyses have identified specific mutations and differentially expressed genes associated with maduramicin and diclazuril resistance, including ABC transporters and folate biosynthesis pathway enzymes (Yu et al., 2025; Zhao et al., 2024). By targeting the non-replicating, environmentally exposed oocyst stage, PBEO-derived compounds may offer a resistance-resilient approach to coccidiosis control, as the lack of replication during sporogony reduces the selective pressure that drives resistance evolution in intracellular stages. Recent epidemiological surveys further confirm the persistent prevalence of *E. tenella* in backyard and commercial flocks across South Asia (Khan et al., 2025), reinforcing the need for novel control options. In parallel, alternative anticoccidial strategies are actively being explored, including the use of biosynthesized zinc nanoparticles (Thagfan et al., 2025) and the application of natural plant extracts evaluated in *in vitro* and *in vivo* systems (Abbas et al., 2025). Our PBEO-targeted, mannitol-cycle approach complements these efforts by providing a defined molecular target rationale for the use of plant-derived essential oils against the environmental sporulation stage of *Eimeria*.

The absence of this pathway in mammals, combined with its conservation across multiple apicomplexan parasites (*Eimeria, Toxoplasma, Cryptosporidium*), suggests that PBEO‑derived compounds targeting the mannitol cycle could have broader antiparasitic applications beyond avian coccidiosis. However, we acknowledge that the mannitol cycle in *Toxoplasma* and *Cryptosporidium* has not been as extensively characterized as in *Eimeria*, and the expression of these enzymes during their respective sporulation stages requires experimental validation.

## Limitations of the study

The findings reported here should be interpreted with the inherent limitations of an *in-silico* study in mind. First, all binding affinities and interaction patterns presented in this work are computational predictions generated by blind molecular docking and should not be regarded as direct evidence of enzyme inhibition. Docking estimates the most thermodynamically favourable pose and approximate binding free energy of a ligand within a static or partially flexible receptor model, but it does not capture the full conformational dynamics of the protein, the role of explicit water molecules, the contribution of cofactors (e.g., NADH/NADPH for M1PDH or ATP for hexokinase), or the kinetics of substrate turnover. Second, the receptor structures used here were either AlphaFold-predicted or refined homology models rather than experimentally determined crystal structures, which introduces a degree of structural uncertainty, particularly in flexible loop regions and at the boundaries of the active sites. Third, our docking experiments were performed against single, isolated enzymes; potential off-target effects on host enzymes, on other *E. tenella* proteins, or on the chicken intestinal microbiota were not assessed. Finally, although the multi-target binding profile observed for several PBEO constituents is consistent with our previously reported in vitro oocysticidal and sporulation-inhibitory activity (Ristanti et al., 2024), a direct mechanistic link between phytochemical binding to the mannitol-cycle enzymes and the observed phenotypic effects on *E. tenella* oocysts has not yet been established. Confirmation of the proposed polypharmacological mechanism will therefore require complementary experimental validation, including (i) recombinant expression and *in vitro* enzymatic inhibition assays of M1PDH, M1Pase and hexokinase using purified PBEO compounds (with nitrophenide as a positive control); (ii) *in vitro* sporulation/oocysticidal assays with isolated lead compounds (e.g., β-selinene, β-caryophyllene and 3-allyl-6-methoxyphenyl acetate); and (iii) *in vivo* efficacy and safety studies in *E. tenella*-challenged broilers. The current docking results are therefore best regarded as hypothesis-generating, providing a rational, structure-based starting point for these next experimental steps.

## Conclusion

This study demonstrates that *Piper betle* L. essential oil phytochemicals bind with high affinity to three key *Eimeria tenella* mannitol cycle enzymes: M1PDH (β-selinene, -7.4 kcal/mol), M1Pase (β-selinene and β-caryophyllene, -5.9 kcal/mol each), and hexokinase (3-allyl-6-methoxyphenyl acetate, -6.2 kcal/mol). KEGG pathway analysis confirms that these enzymes form a non-redundant metabolic unit essential for sporulation, with no bypass reactions annotated in the parasite genome. The multi-target binding profile of PBEO constituents suggests a polypharmacological mechanism that may reduce the likelihood of resistance emergence, a critical advantage given widespread coccidiostat resistance in poultry production. The absence of the mannitol pathway in mammals positions these enzymes as ideal targets for selective intervention with minimal host toxicity. These findings support bio-guided isolation, structure-activity relationship optimization, and *in vivo* efficacy studies of PBEO-derived compounds as transmission-blocking anticoccidial agents targeting the environmental stage of coccidian parasites.

## Ethical approval was not required

This study did not involve human participants or live animals, and therefore ethical approval was not required.

## Declaration of generative AI and AI-assisted technologies in the manuscript preparation process

During the preparation of this work the authors used ChatGPT for sentence-level language polishing only. After using this tool the authors reviewed and edited the content as needed and take full responsibility for the content of the published article.

## CRediT authorship contribution statement

**Syahputra Wibowo:** Writing – original draft, Visualization, Investigation, Formal analysis. **Penny Humaidah Hamid:** Writing – review & editing, Supervision, Funding acquisition, Conceptualization. **Rina Ristanti:** Investigation, Data curation. **Herjuno Ari Nugroho:** Visualization, Software, Formal analysis. **Maulana Malik Nashrulloh:** Software, Formal analysis, Data curation. **Tri Rini Nuringtyas:** Validation, Methodology. **Pamungkas Rizki Ferdian:** Visualization, Investigation. **R. Lia Rahadian Amalia:** Investigation, Data curation. **Tamara Munoz Caro:** Writing – review & editing, Validation. **Nur Balqis Maulydia:** Writing – original draft, Visualization, Investigation. **Shelly Kusumarini:** Writing – review & editing, Data curation. **April Hari Wardhana:** Visualization, Supervision. **Muhammad Cahyadi:** Supervision. **Makoto Matsubayashi:** Validation.

## Declaration of competing interest

The authors declare that they have no known competing financial interests or personal relationships that could have appeared to influence the work reported in this paper.
